# Supplementary material for: Determinants of COVID-19 Vaccine Hesitancy in Portuguese-Speaking Countries: A Structural Equations Modeling Approach
Source: Vaccines (Basel). 2021 Oct 12;9(10):1167. doi: 10.3390/vaccines9101167 (PMC8541102; doi:10.3390/vaccines9101167)
Supplement: Supplementary file 1 [file vaccines-09-01167-s001.zip › vaccines-1384392-supplementary.pdf]

SUPPLEMENTARY MATERIALS

Table S1. Prevalence of vaccine hesitancy for COVID-19 in Portuguese-speaking countries, according to sociodemographic, clinical, pandemic and information consumption characteristics. 2021. (N=6,843).

Variables	n	%	Vaccine Hesitancy		
			n	P (%)	CI95%
**Gender (N=6,805)**					
Male	2015	29.6	344	17.1	15.4-18.8
Female	4,790	70.4	1,100	23.0	21.8-24.2
**Age group**					
18 to 29 years old	2,334	34.1	361	15.5	14.0-17.0
30 to 49 years old	3,295	48.2	722	21.9	20.5-23.4
50 years or more	1,214	17.8	363	29.9	27.3-32-6
**Education (N=6,789)**					
Elementary/High school	1,409	20.8	221	15.7	13.8-17.7
University education	5,380	79.2	1,203	22.4	21.3-23.5
**Government's strategies against the pandemic**					
Disagree	2,146	31.4	435	20.3	18.6-22.0
Agree	4,697	68.6	1,011	21.5	20.4-22.7
**Perceived stress**					
Low	960	14.0	157	16.4	14.1-18.8
High	5,883	86.0	1,289	21.9	20.9-23.0
**Early treatment**					
Never	5,348	78.2	1,118	20.9	19.8-22.0
Rarely	448	6.5	90	20.1	16.5-24.1
Sometimes	719	10.5	163	22.7	19.7-25-9
Often	290	4.2	65	22.4	17.7-27.7
Whenever it comes up	38	0.6	10	26.3	13.4-43.1
**Use of Ivermectin (N=1,495)**					
No	443	29.6	97	21.9	18.1-26.0
Yes	1,052	70.4	231	22.0	19.4-24.6
**Use of Azithromycin (N=1,495)**					
No	1,224	81.9	283	23.1	20.8-25.6
Yes	271	18.1	45	16.6	12.4-21.6
**Use of Chloroquine (N=1,495)**					
No	1,399	93.6	306	21.9	19.7-24.1
Yes	96	6.4	22	22.9	15.0-32.6
**Use of Herbal Medicines (N=1,495)**					
No	1,167	78.1	251	21.5	19.2-24.0
Yes	328	21.9	77	23.5	19.0-28.4
**Fear of Covid-19 repercussions on life (N=6,719)**					
No	712	10.6	82	11.5	9.2-14.1
Yes	6,007	89.4	1,344	22.4	21.3-23.4
**Impact of social distancing on life (N=6,591)**					
Little	754	11.4	150	19.9	17.1-22.9
Moderate	2,256	34.2	496	22.0	20.3-23.8
Severe	3,581	54.3	770	21.5	20.2-22.9
**Had tested positive for COVID-19**					
No	2,018	83.1	479	23.7	21.9-25.7
Yes	411	16.9	88	21.4	17.5-25.7
**History if hospitalization caused by COVID-19**					
No	6,622	96.8	1,396	21.1	20.1-22.1

Yes	221	3.2	50	22.6	17.3-28-7
**Close contact with someone who had Covid-19**					
No	3,255	47.6	650	20.0	18.6-24.4
Yes	3,588	52.4	796	22.2	20.1-23.6
**Close contact with someone who died from COVID-19**					
No	6,070	88.7	1,256	20.7	19.7-21-7
Yes	773	11.3	190	24.6	21.6-27.8
**Conspiracy beliefs about the vaccine**					
The immunity conferred by vaccines against Covid-19 is short-lived	572	8.4	127	22.2	20.0-22.1
Covid-19 vaccines alter DNA	430	6.3	430	100.0	-
The vaccine can cause other diseases, such as autism or autoimmune diseases	1,304	19.1	1,304	100.0	-
COVID-19's vaccine contains chips implanted to control people	944	13.8	944	100.0	-
The vaccine's efficacy and the published studies are false	255	3.7	294	24.7	22.2-27.2

P: Prevalence. n: number. %: percentage. CI95%: Confidence Interval.

Table S2. Adjustment indicators of the latent variable measurement models according with general population, gender, age, and education strata in Portuguese-speaking countries. 2021. (N=6,843).

Indicators	CIR	MIS (CB ↔GB)	CMM
**Model 0: general population**			
RMSEA			
Index	0.029	0.052	0.027
90%CI	0.024 - 0.034	0.050 - 0.054	0.026 - 0.029
CFI	0.983	0.994	0.995
TLI	0.971	0.992	0.995
r[a]			
CB ↔GD	-	0.949	0.948
CIR MIS	-	-	-0.048
**Model 1.1: men**			
RMSEA			
Index			0.032
90%CI			0.029 - 0.035
CFI			0.994
TLI			0.993
r[a]			
CB ↔GD			0.936
CIR MIS			-0.108
**Model 1.2: women**			
RMSEA			
Index			0.025
90%CI			0.023 - 0.037
CFI			0.996
TLI			0.995
r[a]			
CB ↔GD			0.928
CIR MIS			-0.036
**Model 2.1: 18 to 29 years**			
RMSEA			
Index			0.032
90%CI			0.029 - 0.035
CFI			0.994
TLI			0.993

r[a]	
CB ↔GD	0.852
CIR MIS	-0.137
**Model 2.2: 30 to 49 years old**	
RMSEA	
Index	0.028
90%CI	0.025 - 0.030
CFI	0.995
TLI	0.994
r[a]	
CB ↔GD	0.919
CIR MIS	-0.035
**Model 2.3: 50 years or more**	
RMSEA	
Index	0.034
90%CI	0.030 - 0.038
CFI	0.993
TLI	0.991
r[a]	
CB ↔GD	0.924
CIR MIS	0.091
**Model 3.1: Elementary and High school**	
RMSEA	
Index	0.028
90%CI	0.024 - 0.032
CFI	0.996
TLI	0.995
r[a]	
CB ↔GD	0.903
CIR MIS	-0.049
**Model 3.2: University education**	
RMSEA	
Index	0.037
90%CI	0.034 - 0.039
CFI	0.988
TLI	0.987
r[a]	
CB ↔GD	0.822
CIR MIS	-

CIR: COVID-19 individual responses. CB: conspiracy beliefs. GB: general beliefs. MIS: COVID-19 misinformation. CMM: correlated measurement model. RMSEA: root mean square error of approximation. 90%CI: 90% confidence interval. TLI: Tucker-Lewis index. CFI: comparative fit index. [a] Residual correlation (↔) between latent variables.
